# Anemia: A significant cardiovascular mortality risk after ST-segment elevation myocardial infarction complicated by the comorbidities of hypertension and kidney disease

**DOI:** 10.1371/journal.pone.0180165

**Published:** 2017-07-27

**Authors:** Wei-Chieh Lee, Hsiu-Yu Fang, Huang-Chung Chen, Chien-Jen Chen, Cheng-Hsu Yang, Chi-Ling Hang, Chiung-Jen Wu, Chih-Yuan Fang

**Affiliations:** Division of Cardiology, Department of Internal Medicine, Kaohsiung Chang Gung Memorial Hospital, Chang Gung University College of Medicine, Kaohsiung, Taiwan, Republic of China; Azienda Ospedaliero Universitaria Careggi, ITALY

## Abstract

**Background:**

The effect of anemia on patients with ST-segment elevation myocardial infarction (STEMI) remains a controversial issue. The aim of this study was to explore the effect of anemia on STEMI patients.

**Methods and results:**

From January 2005 to December 2014, 1751 patients experienced STEMI checked serum hemoglobin initially before any administration of fluids or IV medications. 1751 patients then received primary percutaneous intervention immediately. A total of 1388 patients were enrolled in the non-anemia group because their serum hemoglobin level was more than 13 g/L in males, and 12 g/L in females. A total of 363 patients were enrolled in the anemia group because their serum hemoglobin level was less than 13 g/L in males, and 12 g/L in females. Higher incidences of major adverse cerebral cardiac events (22.9% vs. 33.8%; p<0.001) were also noted in the anemia group, and these were related to higher incidence of cardiovascular mortality (6.5% vs. 20.4%; p<0.001). A higher incidence of all-cause mortality (8.6% vs. 27.7%; p<0.001) was also noted in the anemia group. A Kaplan-Meier curve of one-year cardiovascular mortality showed significant differences between the non-anemia and anemia group in all patients (P<0.001), and the patients with hypertension (P<0.001), and chronic kidney disease (CKD) (P = 0.011).

**Conclusion:**

Anemia is a marker of an increased risk in one-year cardiovascular mortality in patients with STEMI. If the patients have comorbidities such as hypertension, or CKD, the effect of anemia is very significant.

## Introduction

According to previous reports, a high prevalence (16.9%) of patients with ST-segment elevation myocardial infarction (STEMI) had some degree of anemia at presentation. [1] Anemia is associated with a significantly increased prevalence of baseline comorbidities, and a lower use of guidelines-based therapies, and is associated with increasing odds of in-hospital mortality. [2] The anemic patients have a high prevalence of hypertension, [3] diabetes mellitus, [4] chronic kidney disease (CKD), [5] and heart failure. [6] Many studies already have explored the poor prognosis of anemic STEMI patients. [7, 8] Anemia also influences the incidence of acute kidney injury (AKI) after percutaneous coronary intervention (PCI). [9] Anemia has the potential to worsen myocardial ischemic insult by decreasing the oxygen content of the blood supplied to the jeopardized myocardium [10] and by increasing myocardial oxygen demand through necessitating a higher cardiac output to maintain adequate systemic oxygen delivery. [11] Mixed comorbidities in anemic patients may influence their short-term and long-term mortality. There are few studies that focus on which specific comorbidity could be influenced by anemia in STEMI patients. Anemia seems to be a significant factor related to improving the long-term survival of STEMI patients.

Our study investigates the effect of anemia on long-term cardiovascular mortality of patients with STEMI in the Asian population, and explores which comorbidities could be influenced by anemia.

## Materials and methods

### Patients and groups

From January 2005 to December 2014, 1751 patients experienced STEMI and checked serum hemoglobin initially before any administration of fluids or IV medications. 1751 patients then received primary PCI at our hospital immediately. According to the WHO classification of anemia as a hemoglobin level <13 g/L in men, and <12 g/L in women, [12] the patients were divided into two groups: the anemia group and the non-anemia group.

### Ethics statement

The Institutional Review Committee on Human Research at our institution (Chang Gung Medical Foundation Institutional Review Board) approved the study protocol. We obtained verbal consent by telephone and performed retrospectively analysis.

### Definitions

Our myocardial infarction (MI) definitions are in accordance with the most recent universal definition of MI. [13] Advanced heart failure is defined, according to the New York Heart Association Classification, as being in a class greater than III. Target vessel revascularization (TVR) is defined as any repeat PCI in a target vessel or coronary artery bypass graft (CABG) in a target vessel for the lesions with a diameter stenosis ≧ 70%. [14] The target vessel is defined as the entire major coronary vessel proximal and distal to the target lesion, which includes upstream and downstream branches and the target lesion itself. [14] Cardiovascular mortality is defined as death related to a cardiogenic shock, cardiac arrhythmia, sudden cardiovascular collapse related to possible cardiac reason, and heart failure. All-cause mortality is defined as death from any cause. Major adverse cardiac cerebral events (MACCEs) include an MI, TVR, stroke, and cardiovascular mortality.

### Study endpoints

The primary endpoints of our study were the recurrent MIs, TVRs, strokes, and cardiovascular mortalities during the one-year follow-up period. The secondary endpoints were all the causes of mortality, regardless of cause, during the one-year follow-up period.

### Statistical analysis

Data are expressed as a mean ± standard deviation for continuous variables, or as counts and percentages for categorical variables. Continuous variables were compared using an independent sample t or Mann—Whitney U tests. Categorical variables were compared using a Chi-square statistic. Univariate and multivariate cox regression analyses were performed to identify the associations of one-year cardiovascular mortality. Each correlation between the variables is expressed as a hazard ratio with a 95% confidence interval (CI). A Kaplan-Meier curve was performed for one-year cardiovascular mortality in all groups and differently for specific groups. All statistical analyses were performed using SPSS 22.0 (IBM. Corp., Armonk. NY). A p-value of less than 0.05 was considered to be statistically significant.

## Results

### Baseline characteristics (Table 1)

**Table 1:** Patient characteristics of non-anemia and anemia group.

Variables	Non-anemia (N = 1388)	Anemia (N = 363)	P-value
Age (years)	59.07 ± 12.44	69.21 ± 11.20	< 0.001
Male sex (%)	1198 (86.3)	249 (68.6)	< 0.001
BMI (kg/m^2^)	25.66 ± 3.71	25.45 ± 5.01	0.771
Diabetes (%)	440 (31.7)	178 (49.0)	< 0.001
Current smoker (%)	735 (53.0)	118 (32.5)	< 0.001
Hypertension (%)	776 (55.9)	251 (69.1)	< 0.001
Prior MI (%)	92 (6.6)	35 (9.6)	0.053
Prior stroke (%)	84 (6.1)	38 (10.5)	0.005
CKD stage ≧ 3	352 (25.4)	235 (64.7)	< 0.001
ESRD on maintenance hemodialysis (%)	12 (0.9)	42 (11.6)	< 0.001
Advanced heart failure (%)	103 (7.4)	54 (14.9)	< 0.001
SBP (mmHg)	133.57 ± 33.27	126.22 ± 36.83	< 0.001
Killip level ≧ III (%)	282 (20.4)	143 (39.4)	< 0.001
Chest pain-to-ER time (minutes)	212.37 ± 249.68	260.93 ± 159.73	0.006
Door-to-balloon time (minutes)	100.10 ± 83.75	121.56 ± 45.08	< 0.001
Reperfusion time (minutes)	19.84 ± 11.88	19.56 ± 11.50	0.686
Pain-to-reperfusion time (minutes)	312.29 ± 267.96	381.18 ± 287.71	< 0.001
White blood cell count(x10^3^)	11.6 ± 4.0	11.0 ± 5.3	0.020
Hemoglobin (g/L)	15.1 ± 1.4	11.1 ± 1.5	< 0.001
Blood fasting sugar (mg/dL)	151.3 ± 71.5	171.6 ± 104.6	< 0.001
HbA1C (%)	6.95 ± 2.47	7.02 ± 1.90	0.659
Creatinine (except ESRD) (mg/dL)	1.23 ± 1.02	2.48 ± 2.89	< 0.001
Total cholesterol (mg/dL)	186.21 ± 43.32	164.23 ± 42.69	< 0.001
LDL-cholesterol (mg/dL)	119.44 ± 50.20	100.69 ± 36.32	< 0.001
HDL (mg/dL)	42.01 ± 11.49	41.23 ± 12.30	0.273
Troponin-I (ng/mL)	5.28 ± 16.45	10.13 ± 27.93	< 0.001
CK-MB (ng/mL)	24.53 ± 55.04	31.43 ± 63.75	0.047
LVEF (%)	57.77 ± 13.78	55.78 ± 13.74	0.018
Anterior wall infarction (%)	772 (55.5)	178 (49.0)	0.258
Multiple vessel disease	846 (61.0)	264 (72.7)	< 0.001
Left main disease (%)	80 (5.8)	29 (8.0)	0.142
IABP (%)	225 (16.2)	93 (25.6)	< 0.001
ECMO (%)	45 (3.2)	16 (4.4)	0.265
ACEI/ARBs	1208 (87.1)	276 (76.2)	< 0.001
Beta-blockers	1035 (74.6)	218 (60.2)	< 0.001
Statins	1021 (73.6)	209 (57.7)	< 0.001
Post PCI acute kidney injury (%)	89 (6.4)	56 (15.4)	< 0.001
The need of blood transfusion with PRBC > 2 units (%)	0 (0)	47 (12.9)	< 0.001

Data are expressed as mean ± SD or as number (percentage). Abbreviation: MI: myocardial infarction; CKD: chronic kidney disease; BMI: body mass index; ESRD: end stage renal disease; SBP: systolic blood pressure; HbA1C: glycohemoglobin; LDL: low density lipoprotein; HDL: high density lipoprotein; LVEF: left ventricular ejection fraction; ACEI: angiotensin converting enzyme inhibitor; ARB: angiotensin receptor blocker; PRBC: packed red blood cells.

The average age of the non-anemia group was 59.07 ± 12.44 years, and 86.3% were male. The average age of the anemia group was 60.21 ± 11.20 years and 68.6% were male. There was significant difference between the two groups. The non-anemia group was younger and had more males in it. In the anemia group, there was a higher prevalence of diabetes mellitus, hypertension, prior stroke, CKD at a stage greater than three, end stage renal disease, and advanced heart failure. STEMI was more severe in the anemia group as they had a lower systolic blood pressure and higher Killip classification. We also noted longer door-to-balloon times, and longer chest pain-to-reperfusion times in the anemia group. Reperfusion time was similar between two groups. Significantly lower hemoglobin levels were noted in the anemia group (15.1 ± 1.4 g/L vs. 11.1 ± 1.5 g/L; p<0.001). The anemia group exhibited higher blood fasting sugar levels, higher serum creatinine levels, and higher cardiac biomarkers. In addition to this, they had lower lipid profiles and lower left ventricular ejection fractions. Similar infarcted territory, but more multiple vessel coronary artery disease was noted in the anemia group. The need for intra-aortic balloon pumping was high in the anemia group due to poorer hemodynamic conditions. Poor post-MI medication use because of lower angiotensin converting enzyme inhibitor (ACEI)/angiotensin receptor blocker (ARB) use, lower B-blocker use, and lower statin use was noted in the anemia group. A higher incidence of post-PCI AKI presented in the anemia group, as did the need for blood transfusion, which was only observed in this group, and the need for more than two units of packed red blood cells was 12.9%. Complete database presented in S1 Dataset.

### One-year clinical outcomes (Table 2)

**Table 2:** One-year clinical outcomes of non-anemia and anemia group.

Variables	Non-anemia (N = 1388)	Anemia (N = 363)	P-value
MACCE (%)	276 (22.9)	97 (33.8)	< 0.001
Target-vessel revascularization (%)	173 (15.4)	33 (14.7)	0.840
Recurrent myocardial infarction (%)	51 (4.5)	16 (7.0)	0.130
Stroke (%)	26 (2.3)	10 (4.4)	0.110
Cardiovascular mortality (%)	77 (6.5)	56 (20.4)	< 0.001
Cardiogenic shock and heart failure	49/77 (63.6)	24/56 (42.9)	
Ventricular arrhythmia	14/77 (18.2)	18/56 (32.1)	
Sudden cardiovascular collapse	14/77 (18.2)	14 (25.0)	
All-cause mortality (%)	104 (8.6)	84 (27.7)	< 0.001

Data are expressed as number (percentage). Abbreviation: MACCE: major adverse cardiac cerebral event.

The incidence of MACCEs was higher in the anemia group (22.9% vs. 33.8%; p<0.001). The incidence of TVR (15.4% vs. 14.7%; p = 0.840), incidence of recurrent MI (4.5% vs. 7.0%; p = 0.130), and the incidence of stroke (2.3% vs. 4.4%; p = 0.110) were similar between the two groups. The incidence of cardiovascular mortality (6.5% vs. 20.4%; p<0.001) was higher in the anemia group. In both group, most reasons of cardiovascular mortality were cardiogenic shock and heart failure (non-anemia vs. anemia; 63.6% vs. 42.9%). In addition, increasing possibilities of fatal arrhythmia including definite ventricular arrhythmia and sudden cardiovascular collapse were noted in anemia group (total 57.1%). The incidence of all-cause mortality (8.6% vs. 27.7%; p<0.001) was higher in the anemia group. Complete database presented in S1 Dataset.

### Univariate and multivariate cox regression analyses regarding one-year cardiovascular mortality in all patients (Tables 3 and 4)

**Table 3:** Univariate cox regression analyses about one-year cardiovascular mortality for all patients.

Variables	Hazard ratio	95% CI
Age (years)	1.057	1.042–1.072
Female sex (%)	1.976	1.356–2.878
BMI (Kg/m^2^)	0.972	0.924–1.022
Diabetes (%)	1.799	1.280–2.527
Hypertension (%)	1.201	0.845–1.705
Prior MI (%)	1.021	0.536–1.946
CKD stage ≧ 3 (%)	5.287	3.659–7.640
Advanced HF (%)	3.061	2.017–4.645
Anemia (%)	3.353	2.376–4.731
Anterior wall (%)	1.659	1.160–2.371
Troponin-I (ng/mL)	1.008	1.004–1.012
Killip III, IV (%)	10.132	6.932–10.132
LVEF (%)	0.941	0.928–0.954
Door-to-balloon time (minutes)	1.002	1.001–1.003
Pain-to-reperfusion time (minutes)	1.001	1.000–1.001
Left main disease (%)	2.873	1.768–4.670
MVD (%)	2.857	1.837–4.445
Distal embolization (%)	2.835	1.488–5.402
Post-PCI stenotic severity (%)	1.022	1.010–1.034
DESs implantation (%)	0.402	0.238–0.677
Post-PCI AKI (%)	13.008	9.222–18.349
ACEI/ARB use (%)	0.104	0.074–0.148
B-blocker (%)	0.151	0.104–0.218
Statins use (%)	0.134	0.092–0.197

Abbreviation: CI: confidence interval; BMI: body mass index; MI: myocardial infarction; CKD: chronic kidney disease; HF: heart failure; LVEF: left ventricular ejection fraction; MVD: multiple vessel disease; DES: drug-eluting stent; AKI: acute kidney injury; ACEI: angiotensin converting enzyme inhibitor; ARB: angiotensin receptor blocker.

**Table 4:** Multivariate cox regression analyses about one-year cardiovascular mortality for all patients.

Variables	Hazard ratio	95% CI	P value
Female sex (%)	2.098	1.205–3.653	0.009
Anemia (%)	1.776	1.068–2.952	0.027
Troponin-I (ng/mL)	1.006	1.000–1.012	0.038
Killip III, IV (%)	3.043	1.653–5.603	< 0.001
LVEF (%)	0.966	0.949–0.984	< 0.001
Post-PCI AKI (%)	5.880	3.255–10.625	< 0.001
ACEI/ARB use (%)	0.443	0.249–0.787	0.006
B-blocker (%)	0.405	0.230–0.715	0.002
Statins use (%)	0.407	0.247–0.678	0.027

Abbreviation: CI: confidence interval; LVEF: left ventricular ejection fraction; PCI: percutaneous coronary intervention; AKI: acute kidney injury; ACEI: angiotensin converting enzyme inhibitor; ARB: angiotensin receptor blocker.

The univariate Cox regression analyses identified the following factors as presenting strong significant differences: CKD stage ≧ 3 (HR: 5.287, 95% CI: 3.659–7.640, p<0.001); advanced heart failure (HR: 3.061, 95% CI: 2.017–4.645, p<0.001); anemia (HR: 3.353, 95% CI: 2.376–4.731, p<0.001); Killip classification III, IV (HR: 10.132, 95% CI: 6.932–10.132, p<0.001); left main disease (HR: 2.873, 95% CI: 1.768–4.670, p<0.001); multiple vessel disease (MVD) (HR: 2.857, 95% CI: 1.837–4.445, p<0.001); distal embolization (HR: 2.835, 95% CI: 1.488–5.402, p = 0.002); post-PCI AKI (HR: 13.008, 95% CI: 9.222–18.349, p<0.001); ACEI/ARB use (HR: 0.104, 95% CI: 0.074–0.148, p<0.001).

The multivariate cox analysis revealed that the following factors were independently associated with one-year cardiovascular mortality: female gender (HR: 2.098, 95% CI: 1.205–3.653, p = 0.009); anemia (HR: 1.776, 95% CI: 1.068–2.952, p = 0.027); high serum troponin-I (HR: 1.008, 95% CI: 1.000–1.012, p = 0.038); Killip classification III, IV (HR: 3.043, 95% CI: 1.653–5.603, p<0.001); better left ventricle ejection fraction (LVEF) (HR: 0.966, 95% CI: 0.949–0.984, p<0.001); post-PCI AKI (HR: 5.880, 95% CI: 3.255–10.625, p<0.001.

### The Kaplan-Meier curves of one-year cardiovascular mortality in all groups, and the patients with hypertension, diabetes mellitus, chronic kidney stage ≧ 3, or advanced heart failure (Figs 1–5)

**Fig 1:**
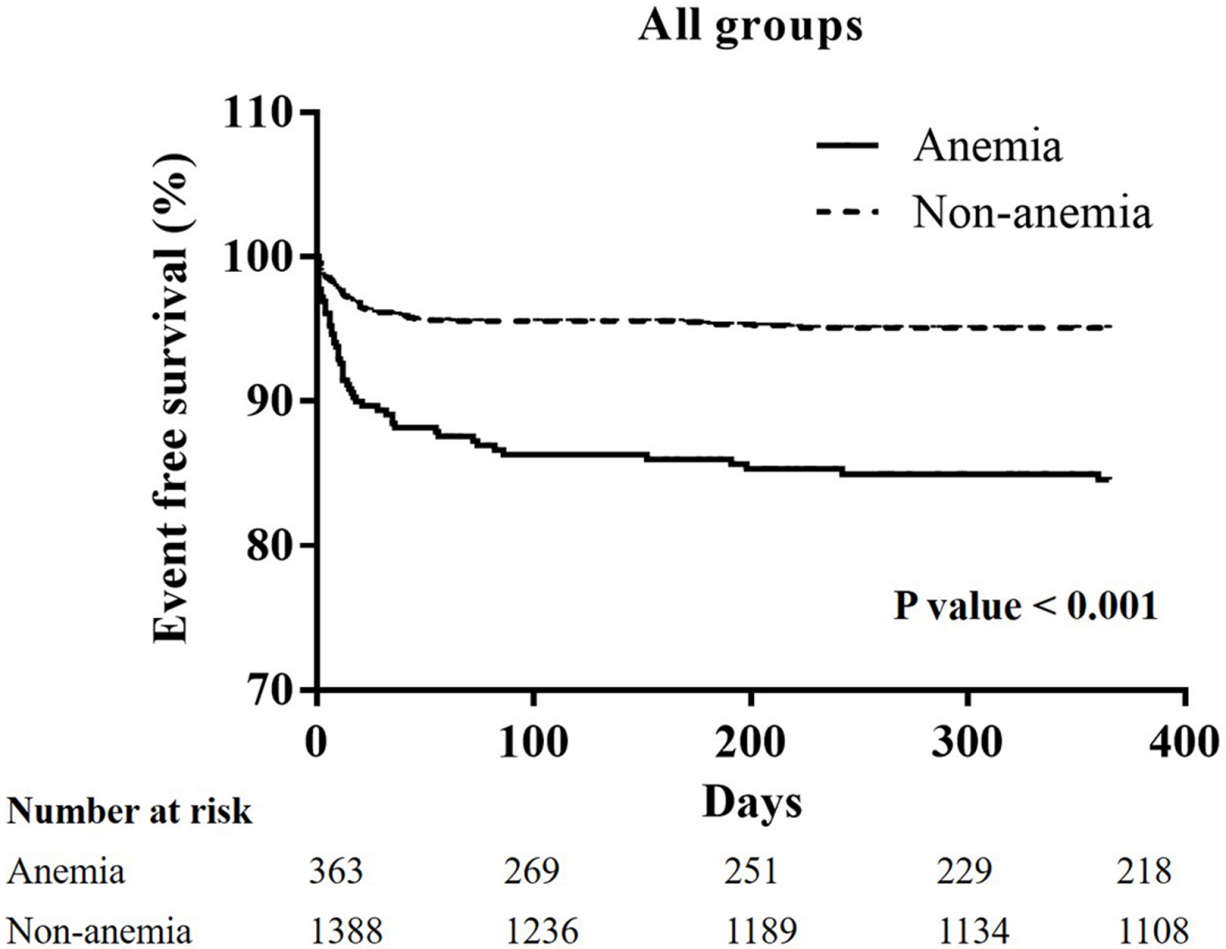
A Kaplan-Meier curve of one-year cardiovascular mortality of all patients: There was a significant difference between the non-anemia group and the anemia group. (p<0.001).

**Fig 2:**
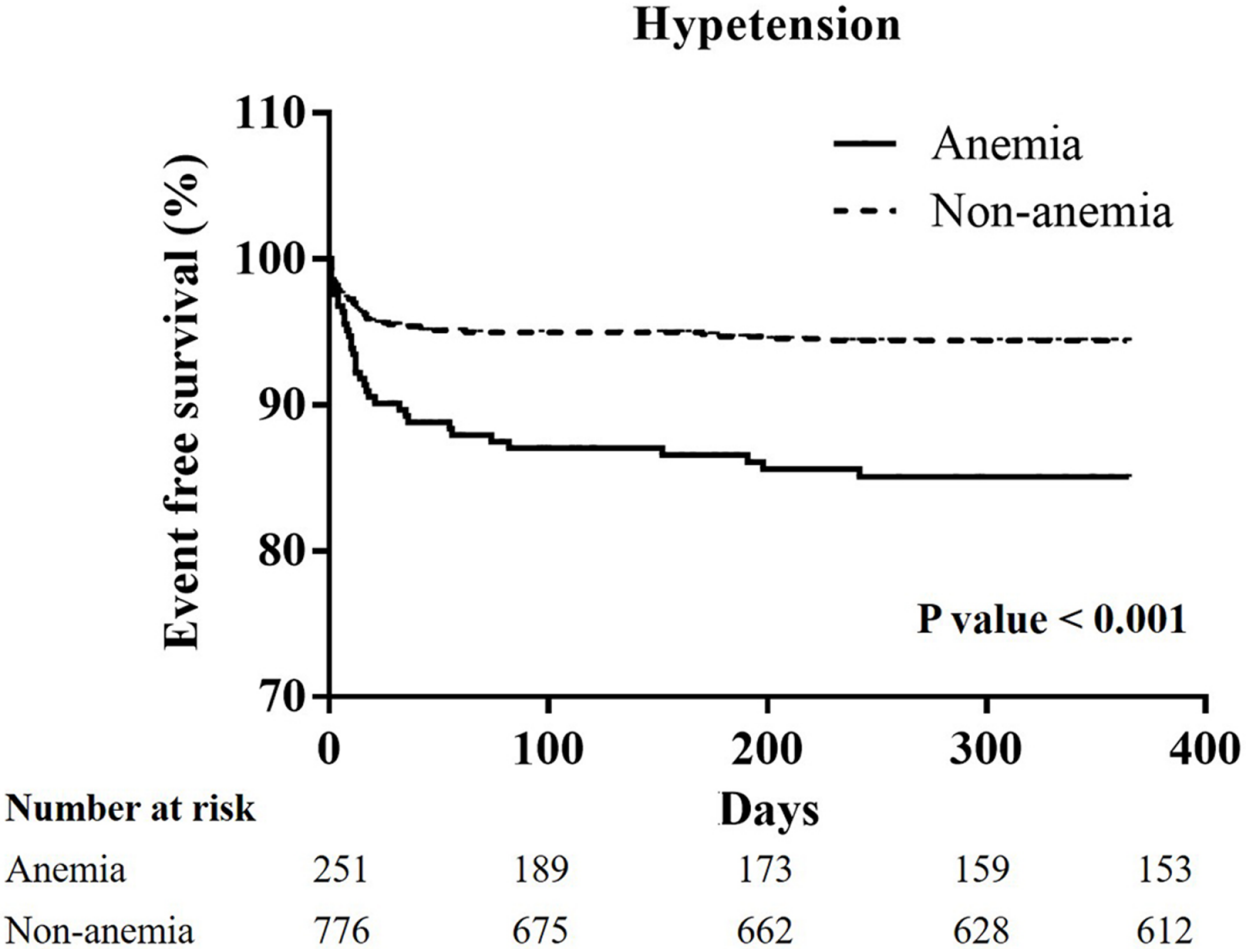
A Kaplan-Meier curve of one-year cardiovascular mortality of STEMI patients with hypertension: There was a significant difference between the non-anemia and anemia group. (p<0.001).

**Fig 3:**
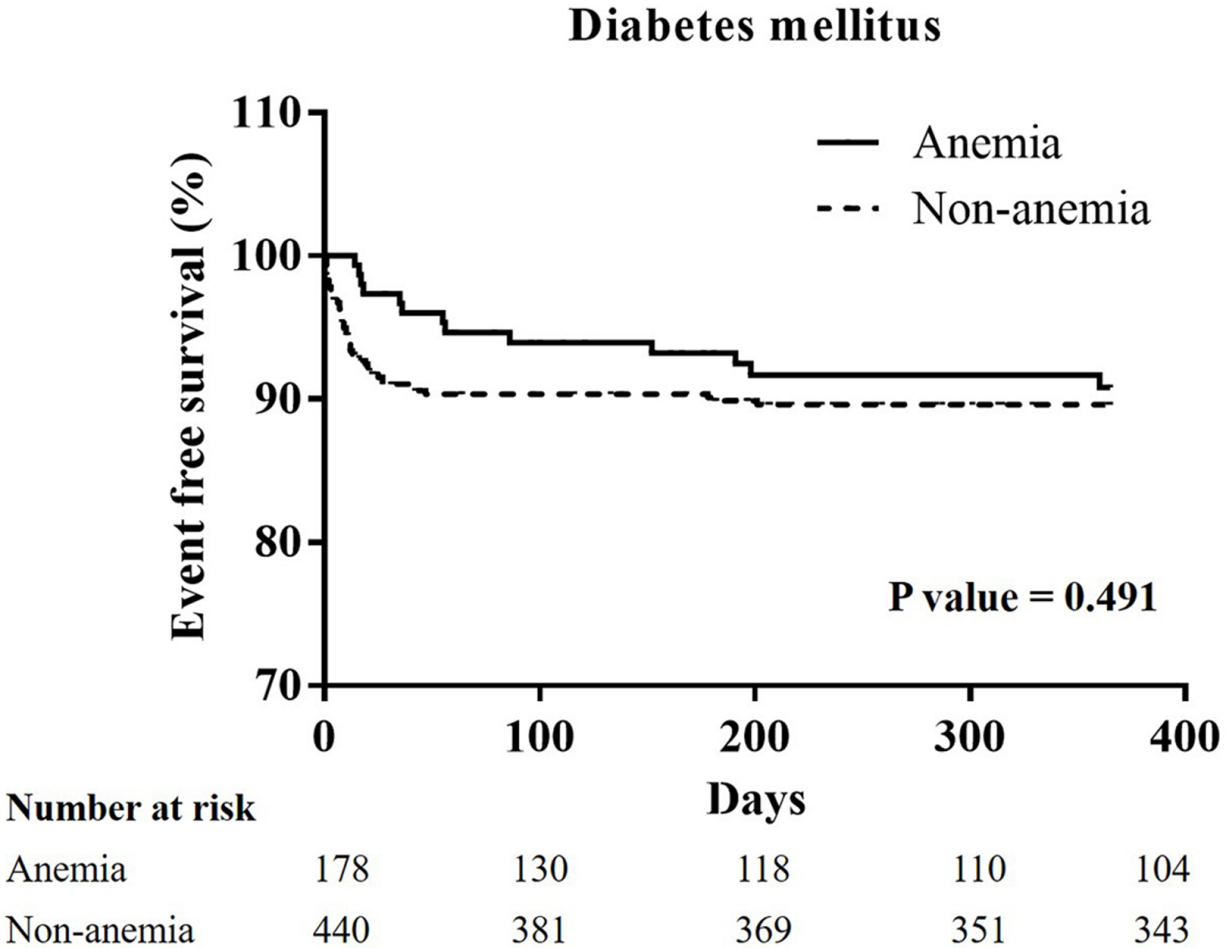
A Kaplan-Meier curve of one-year cardiovascular mortality of STEMI patients with diabetes mellitus: There was no significant difference between the non-anemia and anemia group. (p = 0.491).

**Fig 4:**
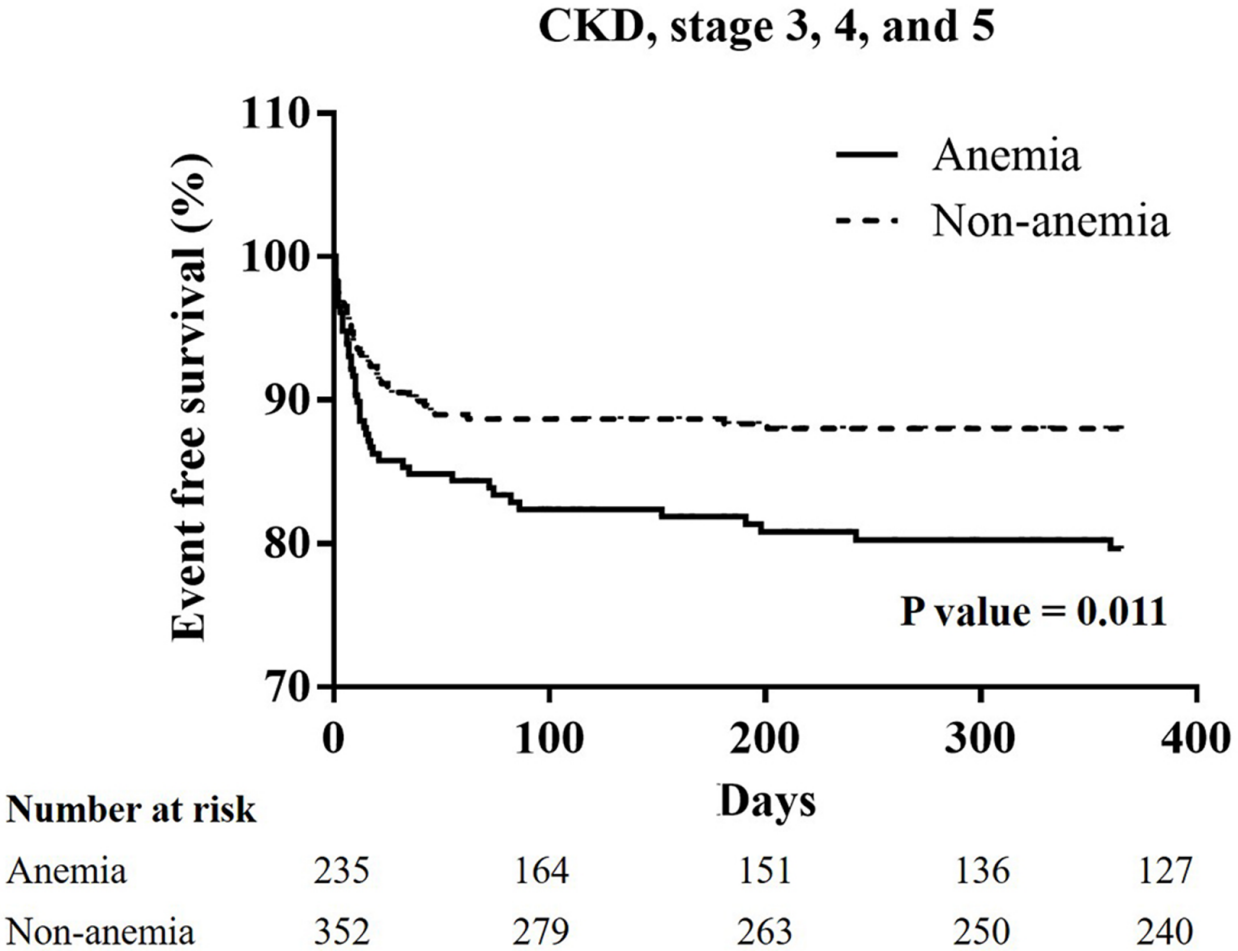
A Kaplan-Meier curve of one-year cardiovascular mortality of STEMI patients with chronic kidney disease stage 3, 4, and 5: There was a significant difference between the non-anemia group and anemia group. (p = 0.011).

**Fig 5:**
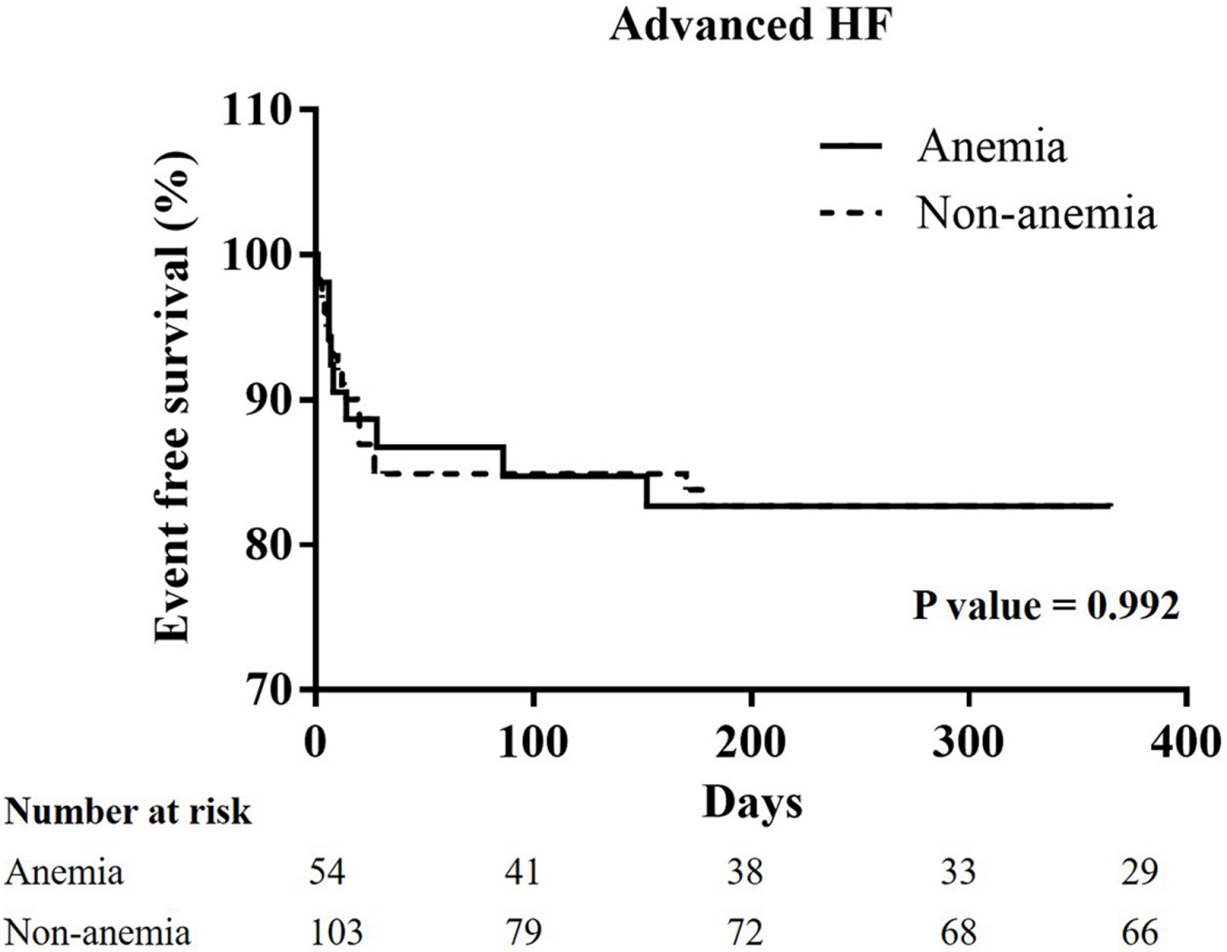
A Kaplan-Meier curve of one-year cardiovascular mortality of STEMI patients with advanced heart failure: There was no significant difference between the non-anemia and anemia group. (p = 0.992).

In all patients, the Kaplan-Meier curve of one-year cardiovascular mortality illustrated better results in the non-anemia group (p<0.001). In the patients with hypertension, or CKD stage ≧ 3, the Kaplan-Meier curve of one-year cardiovascular mortality illustrated better results in the non-anemia group (p<0.001, and p = 0.011; respectively). In the patients with diabetes mellitus or advanced heart failure, the Kaplan-Meier curve of one-year cardiovascular mortality illustrated similar results in the non-anemia group (p = 0.491, and p = 0.992; respectively).

## Discussion

Anemia has been shown high prevalence (around 15%) in the patients with acute MI, and especially in elderly patients (up to 43%). [15] Hemoglobin plays an important role in supplying oxygen to tissues. Shacham et al had indicated that anemia may begin before the patient seeks medical attention, and a longer duration from symptom onset to emergency admission results in a lower admission hemoglobin. They also documented a longer time lag from symptom onset to emergency admission results in a higher level of inflammatory marker. Inflammation was the key in hemoglobin decline during the evolution of STEMI and it emerges before the patient undergoing invasive procedures or IV fluid hemodilution. [16] When hemoglobin decreases, the body may increase cardiac output in order to maintain the normal metabolic demands of tissues. This increases the work load of the heart, and results in myocardial damage. [17] The pathophysiological link between anemia and prolonged QT intervals and increased risk of ventricular arrhythmia is, probably, hypoxia and decreased myocardial oxygen supply. [18] Left ventricular systolic and diastolic dysfunction, increased QTc intervals and QT dispersions and late ventricular potentials were found in patients with beta-thalassemia. [19, 20]

In a previous cohort of patients with STEMI, low hemoglobin was highly statistically significant, and was associated to adverse 30-day cardiovascular outcomes if the baseline hemoglobin dropped below 14 g/L. [21] The worse outcomes observed in anemic STEMI patients might be explained by the theory that anemia decreases oxygen delivery to tissues, and therefore attenuates the ability of collateral flow from nearby patent vessels to limit the extent of myocardial necrosis and peri-infarct ischemia. [22] However, other previous study does not support the fact that anemic patients have a higher one-year mortality. [23] Clearly, the impact of anemia on long-term cardiovascular outcomes is still a controversial issue in anemic STEMI patients. In the Asian population, few studies have focused on this issue concerning cardiovascular outcomes in anemic STEMI patients.

Anemia is more prevalent in the elderly [24] and in patients with multiple comorbidities, such as hypertension, [3] diabetes mellitus, [4] CKD, [5] or heart failure. [6] Anemia is highly prevalent in patients with poor blood pressure control, which also indicates a higher cardiovascular risk and pro-atherosclerotic conditions. [3] Anemia is common among those with diabetes mellitus and CKD, and greatly contributes to patient outcomes. [25] Of hospitalized patients with anemia and heart failure, nearly half experience lower hemoglobin and higher morbidity and mortality. [6] Despite how complex comorbidities contribute to higher mortality in anemic STEMI patients, few studies have focused on the different subgroups in anemic STEMI patients.

In crowded Asian country, most patients who experienced STEMI received primary PCI due to a short transfer time to the PCI center. Previous studies have focused on the predictors of one-year cardiovascular mortality in relation to acute coronary syndrome (ACS) such as age, ACS subtype, and diabetes mellitus, and few studies have focused on only STEMI patients. [26, 27, 28] Early reperfusion changes the clinical outcome of STEMI patients, and we believe that the clinical outcomes are different when STEMI patients are compared to non-ST-segment MI and unstable angina patients due to the different strategies of reperfusion. In this present study, all patients received primary PCI for STEMI. The multivariate analysis of one-year cardiovascular mortality reveals that the independent associations are: female gender; anemia; high serum troponin-I; Killip classification III, and IV; better LVEF; post-PCI AKI; without ACEI/ARB use; without B-blocker use; and without statin use. According to previous studies [2, 9, 27], anemia lowers the use of guidelines-based therapies, and influences the incidence of AKI after PCI. Anemia is therefore an important factor for us consider when devising therapies to improve the long-term outcomes in STEMI patients because we can improve post-PCI AKI and medical care by investigating anemia.

In our study, a total of 363 STEMI patients (20.7%) were defined as the anemia group in accordance with the WHO definition. In the anemia group, patients were elder with a higher prevalence of diabetes mellitus, hypertension, prior stroke, CKD at a stage greater than three, end stage renal disease, and advanced heart failure. In addition, the anemic patients presented with more severe disease, longer door-to-balloon times, longer pain-to-reperfusion times, and poorer medical control. Therefore, a higher incidence of MACCEs was noted in the anemia group, and was contributed by a higher incidence of cardiovascular mortality. In addition, increasing possibilities of ventricular arrhythmia may influence cardiovascular mortality in anemia group. In the patients with hypertension and CKD, worse results of cardiovascular mortality were noted on the survival curve. Anemia is associated with higher cardiovascular risk, and higher blood pressure values in hypertensive patients. [29] The effects of anemia on cardiovascular mortality also presented in the STEMI patients with hypertension. Anemia is common in CKD and has been linked to cardiovascular disease and mortality, especially in advanced stages of CKD. [30] Yacov Shacham et al state that a lower admission hemoglobin level and anemia are independent predictors of post-PCI AKI in STEMI patients. [9] In our study, post-PCI AKI was a strong association of one-year cardiovascular mortality. Shu DH et al state that anemia is not an independent risk factor for 30-day mortality, but that it is associated with reduced survival during the three-year follow-up period in STEMI patients with diabetes mellitus. [4] In our study, a Kaplan-Meier curve of STEMI with or without diabetes mellitus between the non-anemia and the anemia group did not show significant differences. As described in detail previously study, there are some difference such as a lower mean body mass index, developing the illness at a younger age, and early β cell dysfunction in the setting of insulin resistance in the patients developing type 2 diabetes mellitus in East Asian countries when compare to type 2 diabetes mellitus patients in Western countries. [31] East Asian patients with type 2 diabetes mellitus have a higher risk of developing renal complications than Western ones and, with regard to cardiovascular complications, a predisposition for developing strokes. [31] Therefore, anemia may not have a great impact on one-year cardiovascular mortality in the STEMI patients with diabetes mellitus in Asian countries. According to the Reduction of Events by Darbepoetin Alfa in Heart Failure (RED-HF) study, aggressive treatment for anemia did not improve clinical outcomes in patients with systolic heart failure and mild-to-moderate anemia. [32] Anemia is a multifactorial and multidimensional problem. Our study explores the impact of anemia on patients with STEMI in the Asian population, and notes the higher influence on patients with hypertension or CKD.

## Limitations

This was a retrospective cohort study, and we did not randomize our patients to decrease bias. In addition, we only provided and analyzed data from a single center. However, we shared the precious view and results of the clinical outcomes in anemic STEMI patients, especially in the patients with hypertension, diabetes mellitus, CKD stage ≧ 3, or advanced heart failure. Our research also provides insight into possible improvements in healthcare policies for anemic STEMI patients in the future.

## Conclusions

Anemia is a marker of an increased risk in one-year cardiovascular mortality in patients with STEMI. If the patients have comorbidities such as hypertension, or CKD, the effect of anemia becomes very significant.

## Supporting information

S1 Dataset (ZIP)
